# Histopathological study of corneal flap striae following laser *in situ* keratomileusis in rabbits

**DOI:** 10.3892/etm.2015.2171

**Published:** 2015-01-08

**Authors:** LI LIU, FANG-ZHOU SONG, LIAN-YUN BAO

**Affiliations:** 1 Research Center of Molecular Medicine and Cancer, Chongqing Medical University, Chongqing 400016, P.R. China; 2 Ningyi Eye Center, Gulou Hospital Affiliated to Nanjing University Medical School, Nanjing, Jiangsu 210008, P.R. China

**Keywords:** flap striae, laser *in situ* keratomileusis, histopathology, cornea

## Abstract

The aim of the present study was to investigate the histopathological changes and wound healing process of rabbit corneas following conventional laser *in situ* keratomileusis (LASIK) with and without the complication of flap macrostriae. The right eyes of 14 rabbits underwent LASIK with the formation of flap striae (macrostriae group) and the left underwent LASIK alone (control group). Two rabbits were selected at random for sacrifice on days 1, 3, 7 and 14, and at 1, 3 and 6 months postoperatively. The histopathological characters of the corneas were compared by hematoxylin and eosin (H&E), periodic acid-Schiff (PAS) and Masson staining. In the control group, the epithelial basement membrane of the cornea exhibited microstriae and the arrangement of stromal collagen fibers was regular. The width of the microstriae in the flap was 20–40 μm one week after surgery and the microstriae were no longer visible two weeks postoperatively. In the macrostriae group, infiltration of polymorphonuclear cells occurred around the incision and irregular hyperplasia of the epithelium was observed due to undulation of the epithelial basement membrane on the first postoperative day. The collagen fibers and striae of the corneal stroma exhibited irregular undulation one month postoperatively. The area between the corneal flap and stromal bed was distinctly stained by PAS and Masson stains. Macrostriae with a width of 80–120 μm affecting two-thirds of the entire cornea remained visible six months postoperatively. In conclusion, the inflammatory reactions and clinical impact of flap macrostriae were severe. Macrostriae involving two-thirds of the entire cornea remained visible six months postoperatively. Longer-term studies are required to further elucidate the issues associated with corneal flap striae.

## Introduction

Laser *in situ* keratomileusis (LASIK) is currently the most common refractive surgical procedure used for patients with myopia, astigmatism and hyperopia (1,2). In the LASIK procedure, a microkeratome or femtosecond laser is used to create a flap in the corneal epithelium to access the corneal stroma, and the flap is raised to gain access to the underlying stromal tissue. The curvature of the corneal stroma is reshaped by subsequent excimer laser ablation of targeted stromal tissue and the flap is repositioned (3–5). These surgical procedures of LASIK result in faster visual recovery, lower rates of regression and infection, less postoperative pain and better refractive predictability compared with photorefractive keratectomy with complete removal of the central corneal epithelium (6–8).

Despite the advantages of LASIK and incremental advances in the technique, LASIK has certain limitations with regard to risk and visual outcomes, such as anatomic and refractive complications (9). One of the most common complications of LASIK is postoperative striae associated with the creation of the corneal flap, which have a variety of appearances (10). Flap striae are classified into two types, namely macrostriae and microstriae (11). Macrostriae appear as parallel straight lines on retroillumination and are responsible for the reduction of vision (12,13). Visual acuity is usually reduced by two or three lines of microstriae; however, this may be partially improved by a contact lens or artificial tears (14,15). Donnenfeld *et al* investigated the effects of hyperthermia for the treatment of long-standing corneal flap striae following LASIK and found that hyperthermic treatment is a safe, effective treatment option for corneal striae after LASIK (16). Solomon *et al* found that stretching the flap with a cotton-tip applicator is a simple, safe and effective technique for reducing visually significant flap striae (17). However, the formation of striae can lead to a significant loss of corrected visual acuity if the central pupil zone is affected and any later intervention will decrease the probability of successful elimination and visual outcomes (18). Therefore, the sooner that symptomatic deep striae are diagnosed the more promptly effective management procedures can be carried out.

Although there are many viewpoints concerning the causes and pathogenesis of flap striae, such as misalignment or displacement of the corneal flap following flap replacement, movement of the corneal flap and a slippage effect of the corneal flap over the ablated stromal bed following LASIK, the causes and pathogenesis of flap striae have not been definitively confirmed (19–21). In order to study the underlying pathogenesis of flap striae, the present study investigated the histopathological changes in adult New Zealand white rabbit corneas following LASIK with the complication of flap macrostriae by hematoxylin and eosin (H&E), periodic acid-Schiff (PAS) and Masson’s trichrome staining on days 1, 3, 7 and 14, and at 1, 3 and 6 months postoperatively.

## Materials and methods

### Animals

Animal care and use was in accordance with the guidelines established by the Animal Ethics Committee of Chongqing Medical University (Chongqing, China). Fourteen healthy adult New Zealand white rabbits weighing >2 kg were provided by the Experimental Animal Center of Gulou Hospital Affiliated to Nanjing University (Nanjing, China). Animals were housed singly and fed a standard diet *ad libitum*. The right eyes of rabbits were selected to receive conventional LASIK, followed by the creation of flap striae (the macrostriae group) and the left eyes were subjected to conventional LASIK only (the control group).

### Preoperative preparation

During three preoperative days, conventional antibiotic eye drops were applied to the eyes of the rabbits. Each rabbit was generally anesthetized with an intramuscular injection of droperidol (1 mg/kg) and ketamine hydrochloride (50 mg/kg), 15 min preoperatively. Then, 4% oxybuprocaine hydrochloride eye drops were instilled into both eyes of each rabbit for surface anesthesia.

### Surgery

The two eyes of each rabbit were gently proptosed and a hinged corneal flap was cut using a microkeratome (M2; Moria SA, Antony, France), and a spherical ablation of −3.00 diopters (D) was performed on the exposed stromal bed using an excimer laser system (EC-5000 XII; Nidek, Gamagori, Japan) with an energy density of 160–180 mJ/cm^2^ and a 40-Hz pulse rate. Following careful repositioning of the flap, flap striae were generated in the corneal flap in the right eye of each rabbit under a microscope and the direction of the flap was perpendicular to the pedicle. During the surgery, a bandage soft contact lens (radius of curvature, 8.30 mm; diameter, 14 mm) was inserted to prevent flap dislocation.

### Postoperative treatment

During the first week after surgery, the animals received an antibiotic ophthalmic solution for prophylaxis three times daily to mitigate ocular inflammation. The flap margin and adjacent regions were examined under a slit lamp and recorded daily. In this study, the corneal flaps of two eyes in the macrostriae group respectively fell off on the first and third postoperative days. Therefore, these two cases were excluded from the experiments and supplementary animal models were established.

### Histopathological examination

Two rabbits were randomly sacrificed with an intravenous injection of an air overdose into the ear margin on 1, 3, 7 and 14 days and at 1, 3 and 6 months after the LASIK procedure. Then, the eyeballs were removed immediately. The excised corneal tissues were fixed in 10% formaldehyde and embedded in paraffin wax. Serial 3-μm sections were cut and stained with H&E, PAS, or Masson’s trichrome by standard procedures.

## Results

### Clinical observation

#### Eyes of the macrostriae group

**Figure 1:**
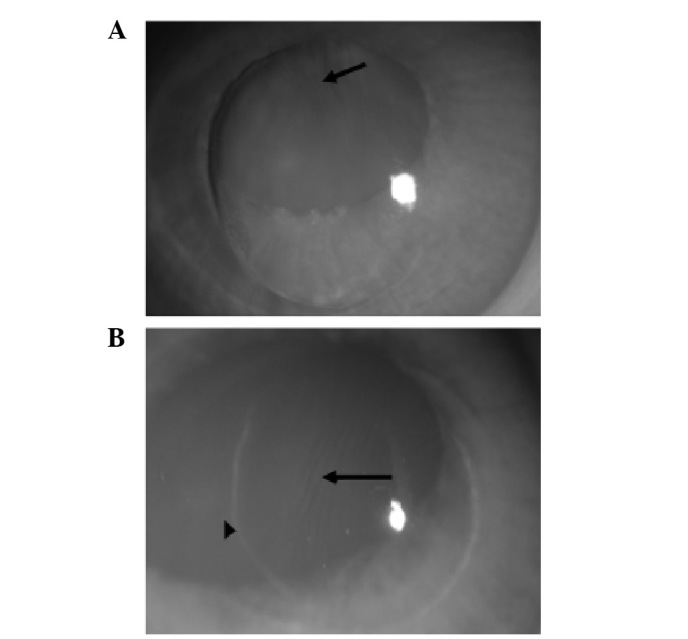
Corneas in the macrostriae group. (A) The flap showed edema and striae (arrow) on the first postoperative day. (B) Striae (arrow) were present on the flap and a partial circle (arrowhead) was visible around the flap one week postoperatively.

On the first postoperative day, edema of the corneal flap was evident and multiple striae were observed. In addition, the corneal stromal bed at the margin of the flap was exposed (Fig. 1A). One week postoperatively, the flap margin was covered by epithelium; multiple striae were present on the flap and a partial ring of opacity was observed around the flap (Fig. 1B). One month after the surgery, the striae on the flap were scarcely visible while the circinate opacity was still present. The striae had largely disappeared three months after surgery and the circinate opacity had almost disappeared six months after the surgery.

#### Eyes of the control group

On the first postoperative day, a transparent corneal flap, evident edema at the flap margin and marginal furrowing of the flap were observed; the epithelium at the margin had not completely grown into the edge of the incision. One week postoperatively, the circinate opacity had recombined with the incision and the corneal epithelium was completely healed with good positioning of the corneal flap and slight edema. One month after the surgery, the edema had completely disappeared and the surface of the flap was smooth; the circinate opacity was fading gradually. The cornea was fully healed and the circinate opacity had disappeared entirely three months subsequent to the surgery. The appearance of the cornea in the control group was similar to that of the normal cornea.

### Histopathological examination

#### Normal corneas

**Figure 2:**
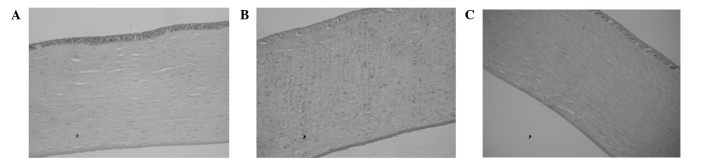
Morphological characteristics of the normal cornea. (A) The corneal epithelium was flat with uniform thickness and the arrangement of stromal collagen fiber was regular (hematoxylin and eosin staining; magnification, ×200); (B) no positive staining of the normal cornea was observed with periodic acid-Schiff stain (magnification, ×200) or (C) Masson’s trichrome stain (magnification, ×200).

A normal cornea stained with H&E is shown in Fig. 2A. The corneal epithelium was flat with uniform thickness. The morphologies of the flat and columnar cells (~4–5 layers) could be observed clearly. The epithelial basement membrane was continuous and flat. The arrangement of stromal collagen fibers was regular and the nuclei of corneal stroma cells exhibited a fusiform shape. In Fig. 2B, a section of normal cornea stained with PAS is shown. Each layer of cells was stained evenly, with the exception of the posterior elastic layer and the endothelium of the cornea, which were stained deeply. A section of normal cornea stained with Masson’s trichrome stain is shown in Fig. 2C. The endothelium, nuclei of corneal stromal cells and endothelial cells were stained red while the remaining tissues were stained blue.

#### Corneas in the macrostriae group

**Figure 3:**
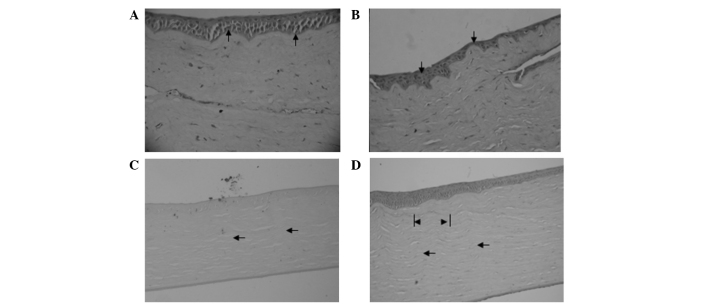
Morphological characteristics of the corneas in the macrostriae group by hematoxylin and eosin staining. (A) Epithelial hyperplasia and ingrowth between the corneal flap and stromal bed, infiltration of polymorphonuclear cells around the incision and undulation of the basement membrane (arrow) were observed on the first postoperative day (magnification, ×400). (B) The thickness of epithelial hyperplasia in the flap was variable (arrow) and the stroma exhibited some striae two weeks postoperatively (magnification, ×400). (C) The corneal stromal collagen fibers and striae had an irregularly undulating appearance with full-thickness striae of the flap, which involved two-thirds of the entire cornea one month postoperatively (magnification, ×200). (D) Three months after surgery, a regularly undulating arrangement of the stromal collagen fibers with a width of 60–80 μm could be observed (magnification, ×200).

Epithelial hyperplasia and ingrowth between the corneal flap and stromal bed were observed on the first postoperative day. The epithelial basement membrane and stromal collagen fibers exhibited an irregularly undulating shape. Infiltration of polymorphonuclear cells around the incision was visible on the first postoperative day (Fig. 3A). On the third postoperative day, the morphological characteristics of the corneas were almost the same as those on the first postoperative day.

One week after surgery, a small number of polymorphonuclear cells had infiltrated around the incision and an epithelial plug was generated at the edge of the corneal flap. Two weeks after surgery, the epithelium was thin (~2–3 layers) in the strial troughs and thick (~7–10 layers) in the strial ridges (Fig. 3B).

One month after surgery, there was no clear difference in the number of epithelial layers between the strial ridge and trough. The corneal stromal collagen fibers and striae presented an irregularly undulating shape, in addition to full-thickness striae of the flap, which affected two-thirds of the entire level of the cornea (Fig. 3C). Three months after surgery, a regular undulating arrangement of the stromal collagen fibers with a width of 60–80 μm was observed (Fig. 3D). In the sixth postoperative month, the morphological characteristics of the corneas were comparable with those in the third postoperative month.

#### Corneas in the control group

**Figure 4:**
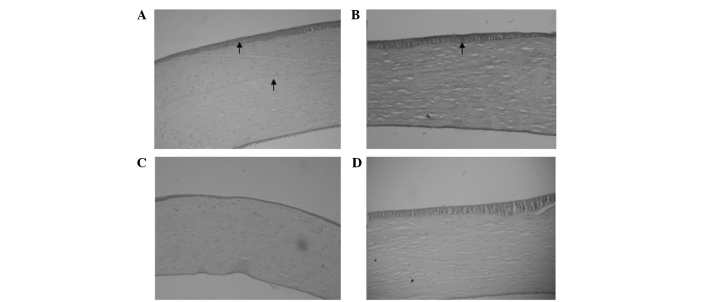
Morphological characteristics of the corneas in the control group. (A) The epithelium of the corneal flap was smooth and the epithelial basement membrane exhibited microstriae of 20–40 μm on the first postoperative day. Hematoxylin and eosin (H&E) staining; magnification, ×200 (B) The microstriae of the epithelial basement membrane became less evident one week after surgery. Masson’s staining; magnification, ×200 (C) The epithelium in the middle of the corneal flap became thinner and the striae on the flap disappeared two weeks after surgery. H&E staining; magnification, ×200 (D) The epithelium and stroma had recovered to normal by the three month after surgery. H&E staining; magnification, ×200.

The epithelium of the corneal flap was smooth and the epithelial basement membrane showed microstriae of width 20–40 μm on the first postoperative day (Fig. 4A). There was no clear difference in the morphological characteristics of the corneas between the third and first postoperative days.

One week after surgery, the microstriae of the epithelial basement membrane became less distinct (Fig. 4B). Two weeks after surgery, the epithelium in the middle of the corneal flap became thinner and the striae on the flap disappeared (Fig. 4C). One month after surgery, there was no evident change in the morphological characteristics of the corneas. Three months after surgery, the epithelium and stroma appeared normal (Fig. 4D).

### PAS and Masson staining of corneas in the control and macrostriae groups

**Figure 5:**
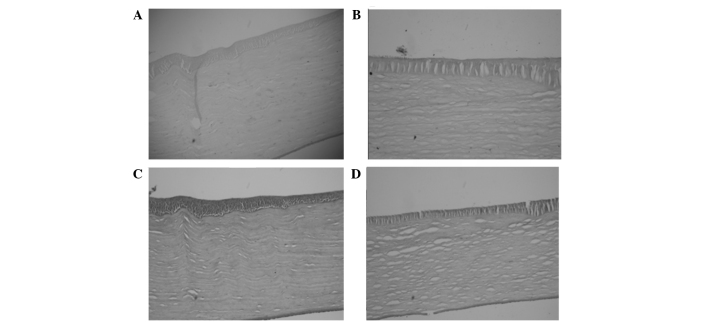
Periodic acid-Schiff (PAS) and Masson’s trichrome staining of the corneas in the macrostriae and control groups, three months after the surgery. (A) The wound margin and the interlayer between the corneal flap and stromal bed were stained in red in the corneas of the macrostriae group (PAS staining; magnification, ×200). (B) PAS staining of a cornea in the control group (magnification, ×400). (C) The area between the flap and stroma was noticeably stained in the corneas of the macrostriae group (Masson’s staining; magnification, ×200). (D) The morphology of the cornea in the control group was comparable with that of the normal cornea (Masson’s staining; magnification, ×200).

There were no significant differences in the PAS and Masson staining of corneas between the macrostriae and control groups three months after the surgery. The flap margin and the interlayer between the corneal flap and stromal bed were PAS stained (Fig. 5A and B). PAS staining is a staining method used to detect glycogen, which indicates that glycogen was generated in the wound healing process of the two groups. Masson staining is used to judge the degree of the lesions and the repair condition of tissues in pathological morphology. The Masson staining procedure stains collagen-rich fibrotic regions blue and muscle red (22). The sections of the cornea were noticeably stained (Fig. 5C and D), which indicates that collagen fibers and muscle fibers were generated in the wound healing process of the cornea.

## Discussion

Although LASIK has become a popular technique for refractive surgery, there are a number of complications that can arise following such procedures, such as interface haze, flap edge scarring, epithelial ingrowth and flap striae or folds (23). Since LASIK surgery and the application of prevention methods for other kinds of complications have become universal, the issues caused by flap striae have drawn an increasing amount of attention from clinicians (18,24). In the present study, the wound healing process and histopathological changes were investigated in adult New Zealand white rabbit corneas following LASIK surgery with the complication of flap macrostriae. It was found that infiltration of polymorphonuclear cells occurred around the incision in the cornea in the macrostriae group on the first postoperative day. Parolini *et al* examined four cases of corneal interface complications that occurred following LASIK and found that severe central inflammation following LASIK could be an extreme manifestation of diffuse lamellar keratitis (25). If foreign bodies are suspected to be the cause of inflammation, early flap lifting with irrigation is imperative for successful treatment (26). The inflammatory reactions of corneas in the macrostriae group were more serious than those in the control group and are likely to influence the clinical outcome of the surgery.

In the control group, the epithelium of the corneal flap was smooth and the epithelial basement membrane showed microstriae 20–40 μm in width on the first postoperative day. Two weeks after surgery, the epithelium in the middle of the corneal flap became thinner and the striae on the flap disappeared. However, in the macrostriae group, the corneal stromal collagen fibers and striae exhibited an irregularly undulating appearance in addition to full-thickness striae of flap, which affected two-thirds of the entire level of the cornea one month postoperatively. Early recognition of the serious postoperative complications of LASIK in order for prompt surgical management to be undertaken is crucial for achieving a successful surgical and visual outcome (27). It has been reported that striae are challenging to eliminate as time goes by since the corneal flap gradually develops fibrosis and lose its original elasticity, which leads to increased resistance to flattening (17,28). Therefore, it is recommended that striae are treated early since delay is likely to cause considerable difficulty (29). Furthermore, the altered arrangement of the corneal stromal collagen fibers and striae in the macrostriae group is likely to increase the adverse effects on visual acuity. Six months after surgery, a regular undulating arrangement of stromal collagen fibers with a width of 60–80 μm remained visible. That is, the clinical impact of the flap macrostriae was prolonged. However, the present study only investigated the time points of 1, 3, 7 and 14 days, and 1, 3 and 6 months postoperatively.

In conclusion, the present study identified that the inflammatory reactions and clinical impact of LASIK were more serious than those in the control group when flap macrostriae were present. The flap microstriae in the control group disappeared two weeks postoperatively. However, macrostriae with a width of 80–120 μm affecting two-thirds of the entire cornea remained present six months postoperatively. Therefore, in order to reduce and prevent the occurrence of flap striae, longer-term studies are required to further elucidate the causes and pathogenesis of flap striae.
